# Die 9. Ausgabe der TNM-Klassifikation nach UICC: Neuerungen für das Fachgebiet Hals-Nasen-Ohren-Heilkunde, Kopf- und Halschirurgie

**DOI:** 10.1007/s00106-026-01724-6

**Published:** 2026-01-16

**Authors:** Christina Sauter, Simon Laban, Thomas K. Hoffmann, Johannes Zenk, Johannes Doescher

**Affiliations:** 1 Klinik für Hals-Nasen-Ohrenheilkunde, Kopf- und Halschirurgie, Universitätsklinikum Augsburg, Sauerbruchstr. 6, 86179 Augsburg, Deutschland; 2 Klinik für Hals-Nasen-Ohrenheilkunde, Kopf- und Halschirurgie, Universitätsklinikum Ulm, Ulm, Deutschland

**Keywords:** Kopf-Hals-Karzinome, Nasopharynxkarzinom, Staging, Kapselüberschreitendes Wachstum, p16, Head and neck neoplasms, Nasopharyngeal neoplasms, Staging, Extranodal extension, p16

## Abstract

**Hintergrund:**

Das seit 1958 existierende TNM-System ist ein etabliertes Instrument zur Klassifizierung solider Tumoren und teilt diese anhand der Tumorgröße und -ausdehnung, des regionalen Lymphknotenbefalls sowie des Vorliegens von Fernmetastasen ein. Die aktuelle 9. Auflage des TNM-Systems bietet umfangreiche Überarbeitungen für den Kopf-Hals-Bereich, insbesondere mit Fokus auf die klinische, radiologische und pathologische extrakapsuläre Ausdehnung von Lymphknotenmetastasen bei entsprechender prognostischer Relevanz.

**Material und Methoden:**

Als Grundlage der Übersichtsarbeit diente die 9. Ausgabe der TNM-Klassifikation der „Union for International Cancer Control“ (UICC). Diese wurde im Juli 2025 veröffentlicht und soll ab Januar 2026 angewendet werden. Die Hintergründe der entsprechenden Änderungen wurden mit einer ausführlichen Literaturrecherche erläutert.

**Ergebnisse:**

Überarbeitungen der TNM-Klassifikation erfolgten wie schon für die 8. Version insbesondere für die Klassifizierung von Lymphknotenmetastasen bei Humanem Papillomavirus (HPV)/p16-positiven Oropharynxkarzinomen hinsichtlich eines kapselüberschreitenden Wachstums. Bei Nasopharynxkarzinomen erfolgte dagegen eine Änderung der UICC-Stadien aufgrund einer besseren prognostischen Aussagekraft. Spezifikationen erfolgten zudem bei der Eindringtiefe von Mundhöhlenkarzinomen. Kleine Speicheldrüsenkarzinome wurden nun zu den großen Speicheldrüsenkarzinomen in das Klassifikationssystem aufgenommen.

**Schlussfolgerung:**

Nach umfangreicher Überarbeitung im Kopf-Hals-Bereich ergeben sich für das Fachgebiet insbesondere relevante Veränderungen hinsichtlich HPV/p16-positiver Oropharynxkarzinome. Inwiefern diese Änderungen sich auch als prognostisch relevant erweisen, bleibt nach Implementierung des neuen TNM-Systems in die klinische Routine abzuwarten.

## Abstract (en)

**Background:**

The TNM system, implemented since 1958, is an established tool for classifying solid tumors and categorizing them based on tumor size and extent, regional lymph node involvement, and the presence of distant metastases. The current 9th edition of the TNM system offers extensive revisions in the head and neck area, with a particular focus on the clinical, radiological, and pathological extracapsular spread of lymph node metastases and the corresponding prognostic relevance.

**Materials and methods:**

The 9th edition of the TNM classification of the Union for International Cancer Control (UICC) served as the basis for the review. It was published in July 2025 and should be implemented from January 2026. The background to the corresponding changes is explained based on a detailed literature search.

**Results:**

As before, revisions to the TNM classification were made particularly for human papillomavirus (HPV)/p16-positive oropharyngeal cancers with regard to extracapsular extension. For nasopharyngeal cancers, there was a modification to the UICC stages due to improved prognostic significance. Specifications were also made for the depth of invasion of oral cavity cancers. Minor salivary gland cancers have now been included in the classification system alongside cancers of major salivary glands.

**Conclusion:**

Following extensive revisions in the head and neck area, there have been notable changes, particularly in the field of HPV/p16-positive oropharyngeal cancer. The extent to which these changes will prove to be prognostically relevant remains to be seen after the new TNM system has been implemented in clinical routine.

Die Grundlage der onkologischen Therapieentscheidungen bei Kopf-Hals-Tumoren basiert auf dem weltweit implementierten TNM-System. In der aktuellen 9. Auflage erfolgt eine Adaptation insbesondere im Hinblick auf die extrakapsuläre Ausdehnung von Lymphknotenmetastasen bei HPV/p16-positiven Oropharynxkarzinomen. Die bisher nach Lokalisation klassifizierten Karzinome der kleinen Speicheldrüsen werden nun analog zu den großen Speicheldrüsenkarzinomen eingeteilt. Zudem wird eine therapierelevante neue Stadieneinteilung bei Nasopharynxkarzinomen zur besseren prognostischen Abgrenzung vorgestellt.

## TNM-Klassifikation im Wandel

Nach Entwicklung der ersten TNM-Klassifikation durch den Franzosen Pierre Denoix zwischen den Jahren 1943 und 1952 wurde die erste Klassifikationsempfehlung der Union for International Cancer Control (UICC) zu Mamma- und Larynxkarzinomen 1958 veröffentlicht [5, 13]. Seither befindet sich die Klassifikation von malignen Tumoren in stetiger Weiterentwicklung mit Aufnahme neuer Entitäten sowie Anpassung an veränderte Prognosen. So findet sich in der aktuellen 9. Ausgabe der TNM-Klassifikation maligner Tumoren eine Implementierung der Karzinome der Nebenschilddrüsen in das TNM-System der Kopf-Hals-Tumoren [4]. Dabei verfolgen die UICC und das American Joint Committee on Cancer (AJCC) das Ziel, Klassifikationen auf Grundlage von publizierten evidenzbasierten Empfehlungen und Datenbankanalysen zu entwickeln, welche dem aktuellen Management von malignen Tumoren entsprechen [3]. Aus diesem Grund finden sich insbesondere für Karzinome des Kopf-Hals-Bereichs zahlreiche Anpassungen, welche im Folgenden genauer erläutert und ab Januar 2026 angewendet werden sollen.

## Extrakapsuläre Ausbreitung bei HPV/p16-positiven Oropharynxkarzinomen

Obwohl die Autoren der aktuellen TNM-Klassifikation „HPV“ und „p16“ gleichsetzen, muss beachtet werden, dass p16 nur als Surrogatparameter für eine HPV-bedingte Genese gilt. Untersuchungen zeigen, dass rund 10–20 % der p16-positiven Karzinome nicht HPV-assoziiert sind [17]. Die im Weiteren gewählte Notation soll diese Problematik explizit berücksichtigen und zwischen Surrogatparameter und direktem HPV-Nachweis differenzieren.

In der Literatur besteht Einigkeit, dass eine extrakapsuläre Ausdehnung (ENE – „extranodal extension“) einen bedeutenden Risikofaktor bei Plattenepithelkarzinomen des Kopf- und Halsbereichs darstellt und mit einem erhöhten Risiko für lokoregionäre Rezidive, Fernmetastasen und einer verminderten krankheitsspezifischen und Gesamtüberlebensrate verbunden ist [16, 18]. Auch in der deutschen S3-Leitlinie für Oro- und Hypopharynxkarzinome wird in diesem Fall bei HPV/p16-positiven Oropharynxkarzinomen der Einsatz einer adjuvanten Radiochemotherapie empfohlen [14]. In der letzten Ausgabe des TNM-Klassifikationssystems der AJCC/UICC wurde ENE jedoch aufgrund der günstigeren Prognose dieser Tumoren und früherer Daten, die auf einen begrenzten prognostischen Wert von ENE in diesem Zusammenhang hindeuten [20], beim nodalen Staging von HPV/p16-positiven Oropharynxkarzinomen nicht berücksichtigt. Eine neuere große multizentrische Studie mit insgesamt 2053 Patienten konnte ENE in der Bildgebung dagegen als den stärksten unabhängigen prognostischen Faktor neben den in Version 8 bereits berücksichtigten Kriterien beim HPV/p16-assoziierten Oropharynxkarzinom für das Gesamtüberleben identifizieren [12]. Darüber hinaus schlugen die Autoren auch eine Höherklassifizierung des N‑Status um einen Rang bei ENE vor. Dies führte zu einer Verbesserung der prognostischen Abgrenzung zwischen den Tumorstadien I und II und übertraf damit die Risikostratifikation des bisher angewendeten TNM-Systems [12]. Dieser Vorschlag wurde in der neuen Auflage der Klassifikation sowohl in der klinisch/radiologischen als auch in der pathologischen Einschätzung umgesetzt. Bei Vorhandensein einer ipsilateralen ENE der Lymphknotenmetastasen ≤ 6 cm erfolgt nun unabhängig von der Anzahl die Einteilung in ein cN2-Stadium, bei bi- oder kontralateralen Lymphnotenmetastasen sowie einer Größe > 6 cm in ein cN3-Stadium. In der histopathologischen Klassifikation stellt ENE nun auch einen integralen Bestandteil der Einordnung dar, wobei ENE-positive Lymphknoten je nach Anzahl in die Stadien pN2 und pN3 klassifiziert werden (Tab. 1). Eine jüngste Validierungsstudie mit fast 15.000 Patienten zeigte, dass die Berücksichtigung von ENE in der aktuellen TNM-Klassifikation gegenüber der vorherigen Einteilung zu einer überlegenen Voraussage hinsichtlich der Therapieergebnisse führte [10].

**Tab. 1:** Definition der klinischen und pathologischen N‑Kategorie HPV/p16-positiver Oropharynxkarzinome.

cN-Kategorie	Bedeutung	pN-Kategorie		Bedeutung
cN0	Keine regionären Lymphknotenmetastasen	pN0		Keine regionären Lymphknotenmetastasen
cN1	Metastase(n) in ipsilateralen LK ≤ 6 cm *ohne *radiologische und/oder klinische ENE	pN1	pN1a	Metastase in einem LK *ohne* ENE
			pN1b	Metastasen in 2–4 LK *ohne* ENE
cN2	Metastase(n) in ipsilateralen LK ≤ 6 cm *mit* radiologischer und/oder klinischer ENE	pN2		1–4 LK *mit* ENE
	Oder			Oder
	Metastase(n) kontra- oder bilateral ≤ 6 cm *ohne* radiologische und/oder klinische ENE			Metastasen in > 4 LK *ohne* ENE
cN3	Metastase(n) kontra- oder bilateral *mit* radiologischer und/oder klinischer ENE Oder Metastase(n) in LK > 6 cm	pN3		Metastasen in > 4 LK *mit* ENE

*LK *Lymphknoten; *ENE* „extranodal extension“

Die ENE wird klinisch, radiologisch oder pathologisch diagnostiziert. Eine klinisch extranodale Ausbreitung ist definiert als das Vorliegen einer Hautbeteiligung, einer Weichteilinvasion mit Fixierung der Raumforderung an benachbarten anatomischen Strukturen oder klinischen Anzeichen einer Nerveninfiltration [3]. Bildgebend zeigt sich ENE bei einer Tumorinvasion durch die Lymphnotenkapsel in entweder perinodales Fett oder benachbartes Gewebe oder durch eine eingeschmolzene Tumormasse, die mindestens zwei benachbarte Lymphknoten mit Verlust ihrer dazwischenliegenden Gewebeschichten und Kapsel umfasst [3, 9]. Dagegen sollte eine pathologische ENE dann diagnostiziert werden, wenn ein Tumor, der innerhalb eines Lymphknotens vorhanden ist, die gesamte Dicke der Lymphknotenkapsel durchdringt und damit in das umgebende Bindegewebe eindringt, wobei eine Stromareaktion nur fakultativ ist. Eine Weichteilmetastase gilt als Lymphknoten mit ENE, wenn sie an einer typischen Stelle eines regionären Lymphknotens liegt [3, 27].

## Lymphknotenquantität der Speicheldrüsenkarzinome

Die bisherige TNM-Klassifizierung für Tumoren der kleinen Speicheldrüsen erfolgte anhand der Lokalisation im Kopf- und Halsbereich analog zu der Einteilung der dort gelegenen Plattenepithelkarzinome. Diese berücksichtigten jedoch nicht die unterschiedlichen Ausbreitungsmuster, die histologische Vielfalt und die klinischen Ergebnisse, die bei malignen Tumoren der kleinen Speicheldrüsen zu beobachten sind [2, 8]. Bei der Klassifizierung und Diagnose von Tumoren der kleinen Speicheldrüsen werden mittlerweile routinemäßig molekulare und immunhistochemische Profile verwendet. Dies hat zur Anerkennung und Neuklassifizierung neuer Entitäten und zu einer präziseren Subtypisierung geführt, was sich auf die Prognose und die therapeutische Entscheidungsfindung auswirkt [24, 26]. Während das TNM-System der UICC auch in der 9. Auflage molekulare Veränderungen noch nicht in die Stadieneinteilung miteinbezieht, werden die malignen Tumoren der kleinen Speicheldrüsen nun analog zu den großen Speicheldrüsen eingeteilt. Regionäre Lymphknotenmetastasen werden jetzt sowohl bei Tumoren der großen als auch der kleinen Speicheldrüsen in c/pN1 klassifiziert bei Metastasen in 1–3 ipsilateralen Lymphknoten ohne ENE und in c/pN2 bei mehr als 3 Metastasen oder ENE. Hieraus resultiert auch eine Stadienanpassung der Speicheldrüsenkarzinome unter Beibehaltung der T‑Klassifikation aus den letzten Versionen (Tab. 2). Diese Änderungen beruhen auf mehreren Analysen, welche die quantitative Lymphknotenlast als einen präziseren prognostischen Faktor für die Überlebensendpunkte ansehen als die bisherige Einteilung nach Größe der Lymphknotenmetastasen [2, 11, 25]. Trotz eines vermuteten fehlenden unabhängigen Einflusses auf die Prognose von ENE bei Berücksichtigung der Lymphknotenanzahl wird dieser Parameter weiterhin in die Klassifikation miteinbezogen [2, 11].

**Tab. 2:** Definition der Tumorstadien der Speicheldrüsenkarzinome

Stadien		T	N	M
0		Tis	N0	M0
I		T1	N0	M0
II		T2	N0	M0
III	IIIA	T3, T4 T1, T2	N0 N1	M0 M0
	IIIB	T1, T2 T3, T4	N2 N1	M0 M0
IV		Jedes T	Jedes N	M1

## Rolle der Eindringtiefe beim Mundhöhlenkarzinom

Die Relevanz der Eindringtiefe (DOI, „depth of invasion“) gegenüber der Tumorgröße beim Mundhöhlenkarzinom wurde bereits in der 8. TNM-Klassifikation anerkannt und in diese integriert [6].

**Abb. 1:**
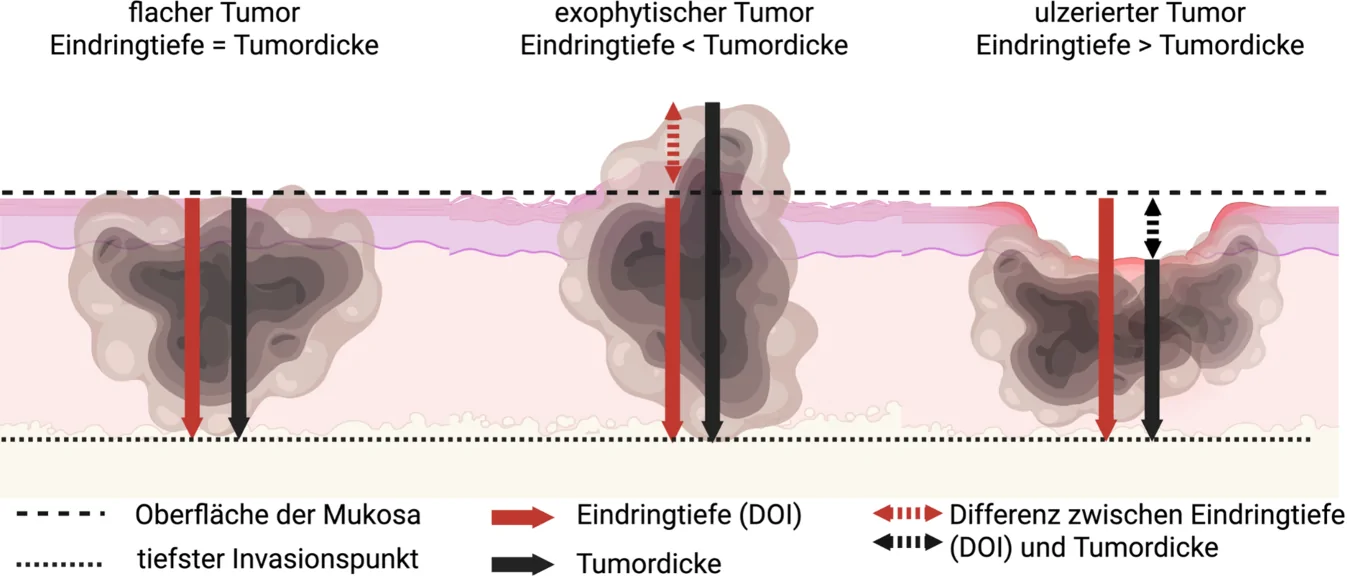
Diskrepanz der Eindringtiefe und der Tumordicke. Adaptiert nach der 9. TNM-Klassifikation der UICC [3]. Created in BioRender. Sauter, C. (2025) https://BioRender.com/56ue73t

Diese Änderungen wurden in der Literatur begrüßt, trotzdem forderten einige Autoren eine aggressivere Behandlung bei Patienten mit einem aufgrund der Tiefeninfiltration bestehenden pT3-Stadium [1] oder die Definition eines pT4-Stadiums bei DOI > 20 mm [15]. Diesen Forderungen kam die UICC nach, indem die DOI und die Tumorgröße in den fortgeschrittenen T‑Stadien nun gemeinsam berücksichtigt werden. Änderungen gibt es hier beim T3- und T4a-Stadium. Das Stadium T3 ist nun definiert als Tumorgröße von 2–4 cm und DOI > 10 mm oder eine Größe > 4 cm und DOI < 10 mm sowie T4a als Tumorgröße > 4 cm und DOI > 10 mm oder Invasion der Kortikalis (mit Beteiligung der Spongiosa/des Knochengewebes) der Mandibula oder Maxilla oder Beteiligung des Sinus maxillaris oder Infiltration der Gesichtshaut. So kann aus einem ehemaligen T3-Stadium (Größe > 4 cm oder DOI > 10 mm) nun bei entsprechender Größe *und* Eindringtiefe ein T4a-Stadium werden. DOI steht in engem Zusammenhang mit anderen histologischen Risikofaktoren, wie der perineuralen Invasion, Lymphknotenmetastasen und einer niedriggradigen Tumordifferenzierung [19, 22]. Die Messung von DOI kann jedoch eine Herausforderung darstellen, da sie präzise histopathologische Verfahren erfordert und sich von der Tumordicke unterscheiden kann, die einfacher zu beurteilen, aber prognostisch weniger aussagekräftig ist ([7]; Abb. 1).

## Stadieneinteilung des Nasopharynxkarzinoms

Die Klassifizierung des Nasopharynxkarzinoms wurde markant verändert. In der 9. Auflage der UICC ist eine fortgeschrittene radiologische ENE nun ein N3-Kriterium, was den negativen Einfluss auf die Prognose widerspiegelt und sicherstellt, dass Patienten mit diesem Merkmal als lokoregionär fortgeschritten eingestuft werden [21, 23]. Bei fehlender Fernmetastasierung wurde auf die Stadien I–III reduziert. Das Stadium I wird aufgrund des unterschiedlichen Risikoprofils in IA (T1‑2 N0) und IB (T1‑2 N1) unterteilt, da Patienten im Stadium IB (T1‑2 N1) eher von einer zusätzlichen Chemotherapie zur Radiatio profitieren [21, 29]. In der neuesten multizentrischen Validierungsstudie mit fast 5000 Patienten von Pan et al. wurden die Überlebensraten von Patienten mit Nasopharynxkarzinom nach der 8. und 9. TNM-Klassifikation direkt miteinander verglichen [21]. Die TNM‑9 erzielt hierbei eine statistisch signifikante Trennung des Gesamtüberlebens (OS) zwischen benachbarten Stadien, beispielsweise zwischen den Stadien I und II (5-Jahres-OS: 96,0 % vs. 93,1 %; *p* < 0,001), welche bei der TNM‑8 nicht signifikant war (96,7 % vs. 95,6 %; *p* = 0,27) [21]. Bei metastasierten Erkrankungen ermöglicht die ausschließliche Einstufung als Stadium IV und die Unterteilung in IVA (M1a, ≤ 3 Läsionen) und IVB (M1b, > 3 Läsionen) individuellere systemische Therapieansätze, einschließlich der Anwendung einer Immuntherapie und palliativer Chemotherapie, wobei sich die Überlebensraten zwischen diesen Untergruppen ebenfalls erheblich unterscheiden ([28]; Tab. 3).

**Tab. 3:** Definition der Tumorstadien der Nasopharynxkarzinome.

		T1	T2	T3	T4
M0	N0	IA	IA	II	III
	N1	IB	IB	II	III
	N2	II	II	II	III
	N3	III	III	III	III
M1	M1a	IVA			
	M1b	IVB			

Folgende weitere Modifikationen wurden in der 9. TNM-Klassifikation vorgenommen. Karzinome unbekannten Ursprungs der Halsregion (CUP, „carcinoma of unknown primary“) werden bei EBV-Positivität analog zum Nasopharynxkarzinom eingeteilt. Bei EBV-CUP beginnt die Stadieneinteilung nun bereits bei Stadium I (T0 N1), sodass insgesamt jeweils eine Herabregulierung der Tumorstadien um einen Rang erreicht wird. Stadium IV bedeutet den Nachweis einer Fernmetastasierung und wird nicht mehr in IVA und IVB unterteilt. Das HPV/p16-positive CUP wird weiterhin analog zur c/pN-Klassifikation des HPV/p16-positiven Oropharynxkarzinoms eingeteilt.

Es wurde erneut hervorgehoben, dass Karzinome des Lippenrots zu den Plattenepithelkarzinomen der Haut des Kopf-Hals-Bereichs gezählt werden. Die TNM-Klassifikation des Schleimhautmelanoms, der Karzinome des Larynx, des HPV/p16-negativen Oropharynx, des Hypopharynx, der Nasenhaupt- und Nebenhöhlen sowie der Schilddrüse bleibt in der 9. Auflage unverändert.

## Fazit für die Praxis

- Die extrakapsuläre Ausdehnung besitzt nun auch bei HPV/p16-positiven Karzinomen prognostische Relevanz.
- Wenn eine Weichteilmetastase an einer typischen regionären Lokalisation für Lymphknoten auftritt, sollte diese als mindestens ein Lymphknoten mit extranodaler Ausbreitung betrachtet werden.
- Bei Karzinomen der kleinen und großen Speicheldrüsen spielt die Anzahl der Lymphknotenmetastasen neben einer extrakapsulären Ausdehnung eine entscheidende Rolle.
- Die Eindringtiefe und die Tumorgröße werden bei Mundhöhlenkarzinomen gemeinsam betrachtet und beeinflussen die Therapieentscheidung.
- Patienten mit Nasopharynxkarzinom profitieren bereits ab dem Stadium IB von einer kombinierten Radiochemotherapie.
- Karzinome unbekannten Ursprungs (CUP) werden je nach EBV- oder HPV/p16-Status wie Naso- oder Oropharynxkarzinome eingeteilt.
